# Assessment of Readiness Toward Flipped Learning Among Novice Nursing Students on Fundamental Nursing Care in Puducherry, South India

**DOI:** 10.7759/cureus.40709

**Published:** 2023-06-20

**Authors:** Kannan Shanmugapriya, Seetha Lakshmi Avudaiappan, Zayabalaradjane Zayapragassarazan, Nagasubramanian Vanitha Rani

**Affiliations:** 1 Department of Medical-Surgical Nursing, College of Nursing, Jawaharlal Institute of Postgraduate Medical Education and Research, Puducherry, IND; 2 Department of Nursing Foundation, Faculty of Nursing, Sri Ramachandra Institute of Higher Education and Research, Chennai, IND; 3 Department of Medical Education, Jawaharlal Institute of Postgraduate Medical Education and Research, Puducherry, IND; 4 Department of Pharmacy Practice, Faculty of Pharmacy, Sri Ramachandra Institute of Higher Education and Research, Chennai, IND

**Keywords:** india, puducherry, fundamental nursing care, nursing students, novice nurses, flipped learning, readiness

## Abstract

Background

This study aimed to assess the readiness toward flipped learning (FL) among novice nursing students in fundamental nursing care in Puducherry, South India.

Methodology

A cross-sectional descriptive study was conducted among 176 first-year B.Sc. Nursing students from three private nursing colleges by purposive sampling technique. In theory and practical classes, the students were taught fundamental nursing procedures such as oral medication, intramuscular injection, peripheral intravenous cannulation, and nasogastric tube feeding as FL. The study was conducted from November 2021 to March 2022. The responses were collected using the Nursing Students' Readiness for Flipped Classroom (NSR-FC) scale with four domains such as personal, technological, environmental, and pedagogical readiness. Pearson correlation and chi-square tests were used to analyze data by incorporating IBM SPSS Statistics for Windows, Version 25.0 (IBM Corp., Armonk, NY, USA).

Results

Among 176 nursing students, 73.9% were aged between 17 and 19 years, the majority (76.7%) were females, and 98% of them used mobile phones to access online FL content. Of the novice nursing students, 69.88% agreed, 27.84% strongly agreed, and 2.27% were in a neutral state for readiness to adopt FL in their nursing curriculum with a mean (standard deviation, SD) value of 77.02 (6.27). Among the four domains of readiness, a positive correlation was found between personal with technological readiness (*P *= 0.001; *r *= 0.446) and pedagogical with personal readiness (*P *= 0.003; *r *= 0.223). Statistically, a significant association was found between students’ readiness toward FL with the occupation of the head of the family, gadgets used to access the FL materials, and Wi-Fi/internet availability in the institutions with a *P*-value <0.05.

Conclusions

The study showed highly positive readiness for FL among nursing students in nursing subjects. It can be leveraged with educational institutions' environmental and technological support by properly utilizing mobile devices, computer laboratories, and access to the internet/Wi-Fi for students from their entry level to enhance FL.

## Introduction

The complexity of the healthcare and learning environment has increased the need for incorporating the latest teaching methods in nursing education to improve knowledge competencies and thereby outcomes of the learner. We are in a position to prepare competent nurses to meet the demands of complex clinical environments [1]. Flipped learning (FL) is a variety of blended learning (BL) in which students learn study materials at home before actual face-to-face lecture class and interact with peers and teachers in the classroom [2]. The FL method is an innovative teaching strategy that improves student knowledge, clinical skills, attitude [3] satisfaction, critical thinking, person-centered care, and competence in real-world situations [4]. Studies have shown that institutions highly recommend nursing students’ usage of gadgets such as mobile phones and laptops to access FL online content due to inadequate technical provisions in their universities [5].

During the COVID-19 crisis, remote teaching became essential, leading to a temporary shift from conventional face-to-face instruction to alternative teaching methods such as video presentations, voice-over PowerPoint slides, and other low-fidelity options. They are the best alternatives for students who cannot access the internet or Wi-Fi in remote areas in developing countries [6]. The shortcomings of the traditional lecture methods are less appealing to current tech-savvy students’ needs as professional courses such as medicine, nursing, dental, and pharmacy are highly inclined toward digital technology and demand self-directed learning [7]. Research conducted in both Western countries and Asian nations has demonstrated the advantages of FL in various aspects, including increased satisfaction, self-directed learning, confidence, clinical skills, personalized learning styles, and improved final exam grades for students [8]. Additionally, studies have highlighted the positive reception of FL by educators, as it facilitates practical application and promotes effective teaching and learning [9].

In Turkey, students with high proficiency in using technological devices were usually more ready to accept FL than those who have inadequate proficiency [10]. Iranian nursing students highly recommended FL as it enhanced metacognitive awareness and readiness for self-directed learning [11]. Technological devices that supported e-learning facilitated FL in nursing education. The undergraduate nursing students from Saudi Arabia expressed above-average satisfaction with all five FL domains: interaction, instruction, instructor, management, and technology [12].

In Sri Lanka, nursing education adopted e-platforms such as Zoom, e-mails, WhatsApp, and Learning Management Systems (LMS) to help bridge the gap in blackboard teaching for clinical training, skill lab, and classroom teaching during the pandemic [13]. The implementation of FL among first-year nursing students resulted in reduced resistance and improved acceptance of this teaching approach. In FL, e-learning served as a pillar of routine classroom teaching and technological devices supported active learning during the COVID-19 situation [14]. The objectives of the study were to determine the level of nursing students' readiness for FL about selected demographic variables and establish a correlation between the personal, technological, environmental, and pedagogical domains with the level of readiness toward FL.

## Materials and methods

A cross-sectional descriptive study was conducted among first-year B.Sc. nursing students enrolled in three private nursing colleges in Puducherry. The study aimed to evaluate the implementation of FL on fundamental nursing care procedures among these students. The quantitative research approach was used. The sample size was calculated based on a previous study, including 20% attrition with a power of 80% and an alpha error of 5% [15]. The three settings had different strengths of student intake per year such as 1,006,030, and the population size was 190. Among them, 14 students were irregular/missed at least one FL. The study involved a sample of 176 participants, who were selected using a non-probability purposive sampling technique. The research was carried out from November 2021 to March 2022. The pilot study was conducted from May to September 2021 and found feasible. The posttest I, posttest II, and posttest III were completed in two, six, and 12 weeks. The purpose and protocol of the study were explained and written informed consent was obtained from the participants. Both the male and female students who underwent all the sessions of FL were included in the study. The students who were irregular/absent from any one session were excluded from the study.

In this study, a self-administered questionnaire titled Nursing Students' Readiness for Flipped Classroom (NSR-FC) was utilized. The questionnaire was specifically designed for nursing students and consisted of 20 items across four domains: personal readiness, technological readiness, environmental readiness, and pedagogical readiness. These domains encompassed various aspects related to students' preparedness for engaging with FL.

The readiness questionnaire utilized a five-point Likert scale, allowing participants to indicate their level of agreement or disagreement using the following response options: strongly disagree, disagree, undecided, agree, and strongly agree. The total score was 100. The Likert scores were interpreted in the study as follows: a score of ≥3 was considered positive, indicating agreement, while a score of ≤2 was considered negative, indicating disagreement. The validity of the tool was established with a comparative fit index of 0.87, and the reliability of the questionnaire was assessed using Cronbach's alpha coefficient, which yielded a value of 0.9 [5]. These indicators suggest a high level of internal consistency and reliability of the questionnaire in measuring the readiness of nursing students for flipped learning. 

Attendance in the FL classes was mandatory for all students, as these four procedures were not taught or demonstrated by their college faculty. To ensure the inclusion of these procedures, special permission was obtained from the subject in-charges in each setting, granting the primary investigator the authority to conduct and oversee the handling of these procedures. At the end of FL, the responses from the students were collected in the classroom. The primary investigator took help from the students' subjects in-charges of each setting to supervise during data collection, which averted contamination among the research participants.

Study procedure

It consists of three phases.

Phase I (Preparatory/Orientation Phase)

This phase of the study lasted for two weeks. Requested permission from the Principal College of Nursing of three private nursing colleges, obtained informed consent from the students, explained the purpose of the study, and created/collected the Gmail accounts of all the participants. Provided students with detailed instructions and orientation on how to use the Google Classroom Application (GCA), which is a free learning software [16]. The fundamental nursing care procedures such as oral medication administration, intramuscular injection, peripheral intravenous cannulation, and nasogastric tube feeding were taught by GCA. The students received instructions via the Google Classroom platform regarding access to instructions one week before the lecture class. The students were instructed to access the online content through gadgets such as smartphones, laptops, and desktops. The primary investigator loaded learning contents in GCA, before the actual face-to-face class for each procedure. It was intimated that the online content will vanish after one week. The students were given reminders via GCA/WhatsApp to view the contents within the due date.

Phase II (Implementation/Teaching Phase)

The duration of this phase was 60 minutes (lecture and skill demonstration) for each procedure. In each setting, one procedure was taught in one month.

Face-to-face theory classes: It included a brief theory class for 30 minutes (as the students had already read the module and seen the video demonstration) on each nursing procedure, doubt clarification, and peer interaction by the face-to-face teaching method.

Laboratory demonstration: It involved a 30-minute demonstration of each procedure in the skill lab for one batch of students (the students had already viewed the procedure video captured by the primary investigator and validated by the experts in the field). Each batch consisted of 15 students and the last two batches consisted of 13 students (a total of 176 students). The theoretical instruction and demonstration of each procedure were conducted on a monthly basis, with one procedure covered each month. This process was carried out consecutively for four months within the given setting, ensuring the completion of all four procedures.

Phase III (Evaluation Phase)

This phase took one week in each setting. The FL readiness responses were collected from the flipped students at the end of teaching after one week.

Data were analyzed using IBM SPSS Statistics for Windows, Version 25.0 (IBM Corp., Armonk, NY, USA). Descriptive statistics (frequency, percentage, mean, and standard deviation [SD]) and inferential statistics (chi-square) were used to find out the relationship between the level of knowledge and background variables.

The Institutional Ethical Clearance certificate was obtained from Vinayaka Missions College of Nursing with IEC number VMCON/IEC2020/01. The participation was voluntary, and written informed consent was obtained from the students.

## Results

Among 176 first-year nursing students, 73.9% were aged between 17 and 19 years and the majority (76.7%) were females, 55.1% of the students were from rural, and 44.9% were from urban residential areas of Puducherry, India. Nearly 50% of the parents were in elementary occupation, agriculture, or fishery work. Of that, 30% had a family income of less than 10,000 Indian rupees per month, and 98% of the students used mobile phones to access online FL content rather than laptops and desktops. Internet connectivity was available in all three institutions but student access was denied. Among the samples, 12.5% were from the hostel, and the remaining were from home.

Teach domains of readiness assessed the following from the flipped students.

Personal readiness (five items)

It focuses on the student’s willingness to engage, utilization of time, interest in gaining learning outcomes, need for hands-on training, and interest to play online quizzes as a classroom activity in FL.

Technological readiness (seven items)

This component deals with the ability to use document viewing software, access to online learning content, and instant message software such as WhatsApp to communicate with peer groups and faculty. It also consists of downloading files from the internet to operate online media players, searching online resources, and convenient handling of mobile phone devices to access online videos.

Environmental readiness (five items)

It recognizes the access to an internet connection, learning resources, technology-enhanced learning practices, availability of technical help, and computer labs from the university to access FL.

Pedagogical readiness (three items)

This type of readiness reflects on the student’s preference for teacher interaction on an individual basis to clarify doubts, convenient use of the online platform to interact with faculty and peer groups, and the importance of student-centered classroom learning processes such as quizzes rather than learning from routine lecture methods.

**Figure 1:**
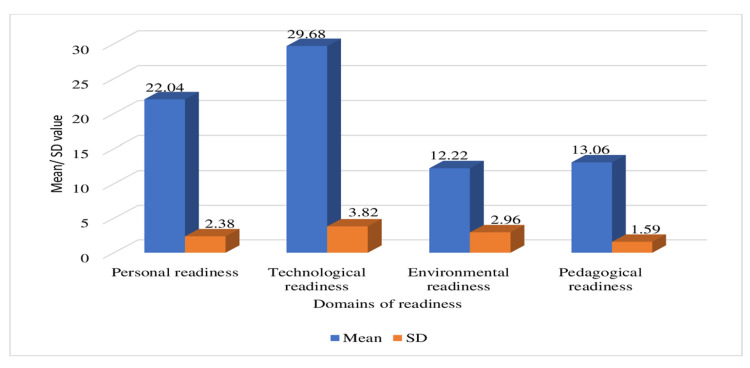
Mean and SD values of four domains of FL readiness. FL, flipped learning; SD, standard deviation

Figure 1 depicts the mean and SD values of the four domains of *FL readiness*, with technological, personal, pedagogical, and environmental readiness. Environmental readiness scored the least, and technological readiness scored the highest among all the four domains.

Table 1 depicts the frequency- and percentage-wise distribution of the level of readiness toward FL among nursing students. In terms of readiness for FL, the majority of the nursing students 123 (69.88%) agreed, 49 (27.84%) strongly agreed, and 4 (2.27%) were found to be neutral.

**Table 1:** Distribution of readiness toward FL among nursing students (N = 176). FL, flipped learning

Level of readiness toward FL	Frequency (*n*)	Percentage (%)
Neutral	4	2.27
Agree	123	69.88
Strongly agree	49	27.84
Mean ± standard deviation	77.02 ± 6.27	

Table 2 shows a positive correlation between personal and technological readiness (*r* = 0.446) at *P *< 0.001, and pedagogical and personal readiness (*r* = 0.223) at *P* < 0.01. No significant positive correlation was found between technological readiness, environmental readiness, and pedagogical readiness.

**Table 2:** Correlation between personal, technological, environmental, and pedagogical readiness among nursing students (N = 176). ^*^P < 0.001 level significance. ^**^P < 0.01. SD, standard deviation

Correlation of readiness	Mean	SD	Pearson *r* value	*P*-value
Personal readiness	22.04	2.38	0.446	0.000*
Technological readiness	29.68	3.82		
Technological readiness	29.68	3.82	0.025	0.739
Environmental readiness	12.22	2.96		
Environmental readiness	12.22	2.96	-0.068	0.368
Pedagogical readiness	13.06	1.59		
Pedagogical readiness	13.06	1.59	0.223	0.003**
Personal readiness	22.04	2.382		

Table 3 shows the frequency- and percentage-wise distribution of readiness toward flipped learning among nursing students. In terms of personal readiness, it was found that 54% of the students agreed and 39.1% strongly agreed to engage in FL. Most of them reported that they achieved learning outcomes through this new technique. The students expressed that they need hands-on training to engage in effective FL and that they were interested in online quizzes as a classroom activity during feedback at the end of the course. The technological readiness revealed that 58% agreed and 33.4% strongly agreed that the students can use GCA to read online materials, 44.9% agreed and 43.8% strongly agreed that they can effectively use instant messaging software such as WhatsApp for communication. Nearly 85% expressed that it was more convenient to use mobile phones than any other gadget to access FL.

**Table 3:** Distribution of NSR-FC (N = 176). NSR-FC, Nursing Students’ Readiness for Flipped Classroom; N, number

NSR-FC	Strongly disagree (1)		Disagree (2)		Undecided (3)		Agree (4)		Strongly agree (5)	
	*n*	%	n	%	n	%	n	%	n	%
Personal readiness										
I am willing to engage in flipped learning.	1	0.6	1	0.6	10	5.7	95	54	69	39.1
I am willing to make the time available for flipped learning.	0	0	2	1.1	9	5.1	85	48.3	80	45.5
I am interested in achieving my learning outcome through flipped learning.	1	0.6	0	0	7	4	93	52.8	75	42.6
I need hands-on training for engaging in a flipped classroom.	0	0	0	0	5	2.8	73	41.5	98	55.7
I am interested in playing online quizzes as a classroom activity.	1	0.6	1	0.6	6	3.4	76	43.2	92	52.3
Technological readiness										
I can use the Google Classroom app to read materials.	1	0.6	1	0.6	13	7.4	102	58	59	33.4
I can use instant messaging software (i.e., WhatsApp) to communicate with people.	1	0.6	2	1.1	17	9.7	79	44.9	77	43.8
I can download files from the internet. (Google Classroom app)	0	0	2	1.1	15	8.5	77	43.8	82	46.6
I can operate the Google Classroom app to watch or listen to the study materials.	2	1.1	5	2.8	14	8	94	53.4	61	34.7
I can search for the information that I need from online resources.	0	0	4	2.3	18	10.2	93	52.8	61	34.7
It is convenient for me to use a computer and/or a mobile phone in my learning.	1	0.6	6	3.4	18	10.2	80	45.5	71	40.3
I have learned and am familiar with learning from video lectures (Google Classroom).	2	1.1	2	1.1	21	11.9	84	47.7	67	38.2
Environmental readiness										
I have access to the internet connection at the college/university (e.g., Wi-Fi).	141	80.2	30	17	1	0.6	2	1.1	2	1.1
My university provides the necessary resources for flipped learning.	116	65.9	32	18.2	4	2.3	21	11.9	3	1.7
My university promotes technology-enhanced learning practices among students.	15	8.5	29	16.5	31	17.6	94	53.4	7	4
Technical help is available for e-learners at the university.	7	4	33	18.8	24	13.6	96	54.5	16	9.1
Computer labs in my institutions are the most important assets for using flipped learning.	68	38.6	23	13.1	19	10.8	49	27.8	17	9.7
Pedagogical readiness										
I prefer a student-teacher interaction on an individual basis (1:1) to clarify doubts.	4	2.3	0	0	3	1.7	86	48.9	83	47.1
It would be convenient if an online platform could be used to interact with teachers and classmates.	0	0	6	3.4	3	1.7	96	54.5	71	40.4
I prefer a student-centered classroom learning process (such as role-play, problem-based learning, debates, and quizzes) rather than learning from a traditional lecture.	1	0.6	5	2.8	7	4	79	44.9	84	47.7

Environmental readiness showed that 80.2% strongly disagreed and 17% disagreed that they were able to access Wi-Fi/internet connection at their college campus and university even though the access is available for the faculty members because access to internet/Wi-Fi by students was denied in the campus. Among them, 64.9% strongly disagreed and 18.2% disagreed that the colleges provided necessary resources for FL access. More than 50% reported that the colleges promoted technology-enhanced learning practices and started providing technical help very recently due to the COVID-19 outbreak. Half of the students agreed that computer labs within their institution were not an important asset for FL, whereas the other half did not agree regarding the same.

Among pedagogical readiness, students preferred student-teacher interaction on an individual basis (1:1) to clarify doubts. About 54.5% expressed that it was convenient if an online platform could be used to interact with the teachers and classmates. The students preferred student-centered classroom learning processes such as role-play, problem-based learning, debates, and quizzes rather than learning from a traditional lecture method.

Table 4 explains the demographic variables such as the occupation of the head of the family (*X*^2^ = 33.53; degrees of freedom [df] = 18), gadgets used to access online materials (*X*^2^ = 13.67; df = 4), and net connection on the college campus (X^2^ =9.80; df = 2) had shown a statistically significant association between the levels of readiness toward FL among nursing students with a *P*-value 0.014, 0.008, and 0.007 (*P *< 0.05), respectively.

**Table 4:** Association between the levels of readiness in selected demographic variables toward flipped learning among nursing students (N = 176). ^*^P < 0.05. ^**^P < 0.01  level significance. df, degrees of freedom; FL, flipped learning

Selected demographic variables	*X*^2^	df	*P*-value
Occupation of the head of the family	33.53	18	0.014^*^
Gadgets used to access FL content	13.69	4	0.008^**^
Availability of Wi-Fi/internet connection	9.8	2	0.007^**^

## Discussion

Flipped learning, otherwise called an inverted classroom, has emerged as an important pedagogical aid among higher education institutions in developing countries like India. It allows active learning in limited class duration under faculty guidance [17]. FL demonstrated several benefits, including improved self-paced learning, self-efficacy, engagement, satisfaction, confidence, competence, learning style, and clinical skills. It also enhanced satisfaction among educators [18,19]. It is the educator’s responsibility to prepare next-generation students to meet the changing healthcare needs [20]. Of the nursing students from the current study, 52.8% agreed and 42.2% strongly agreed that they are interested to achieve learning outcomes through FL. The findings of the study were consistent with a focus group interview conducted in the Kingdom of Bahrain, which indicated that the implementation of the FL method stimulated active learning among students. Furthermore, it enhanced their understanding of concepts and fostered deeper and broader thinking [21].

FL should be implemented carefully in the curriculum based on the number of students, preparation time, course content, faculty competencies, training, and planning [22]. Students expressed that this innovative method improved their self-regulation, co-regulation, academic output, competence, and active participation and relieved learning pressure, which was prominent in routine learning [23]. FL is the best alternative for converted learning among both Chinese and Egyptian students, and it also improved the ability of application, analysis, practical ability, confidence, motivation, and clinical skills among students [24,25]. This study showed that the students are highly engaged and interested in achieving their practical skills in FL. It is consistent with the findings conducted among Chinese nursing students that FL improved a sense of cooperation, team spirit, teamwork, autonomy, communication, expression, thinking ability, analysis capability, learning interest, effective curriculum implementation, resilience, and resolution [26]. Even though the students need to spend more time preparing in FL than in routine learning, they spent less time reviewing the contents in FL than in routine chalkboard learning. The finding is consistent with this study that the students are highly motivated to utilize their free time for FL in their home/hostel [27].

 In this study, personal and technological readiness and pedagogical and personal readiness were highly correlated. This result is parallel with the study conducted in Sri Lanka, which showed a correlation among all four domains [5]. This study showed that the fresher's personal readiness was stronger but they desire hands-on training. This finding was similar to the study conducted in South Africa that concluded that personal readiness is very essential, which conceded effective FL even though there was poor technological and equipment readiness among students [28].

Under technological readiness, the students found it convenient to utilize their mobile phones to access FL content via GCA. This finding was consistent with the study findings among Western Iran nurses that the students improved metacognitive awareness when they learned the online content via the LMS [11]. The students preferred student-centered learning where teachers gave individual attention and taught through different approaches such as quizzes, role-play, and problem-based learning which was absent in routine learning. This result is parallel with the meta-analysis of randomized controlled trials (RCTs) conducted in China, FL enhanced individualized refinement of theoretical knowledge and skill compared to routine chalkboard learning [29].

They expressed that the learning environment in Puducherry should be strengthened by allowing the nursing students to access Wi-Fi/internet and mobile phones to access e-contents. Educational institutions should take steps to provide adequate technological support for blended learning, and existing computer labs should be efficiently used for innovative learning such as blended/FL not only in COVID-19 situations but in the future also.

Among 176 students, nearly 86% were comfortable accessing FL online content via GCA and 100% of them managed with their personal network connection even though the majority of the parents were in elementary occupation. The occupation of the head of the family, devices used to access online materials, and Wi-Fi/internet connection in the institutions had shown a statistically significant association between the levels of readiness toward FL. These findings of the study were supported by a study conducted in Saudi Arabia that technical support is essential to design online content, effective use of online tools, and proper time management [30].

The limitations are that the study was conducted only in private nursing colleges in Puducherry and included four fundamental nursing care procedures only. The recommendations are that similar studies can be conducted as comparative studies among private and government nursing colleges to generalize the findings.

## Conclusions

The study showed that nursing students exhibit a high level of readiness to adopt FL in their curriculum, particularly in nursing subjects that require skill-oriented learning. FL will be further enhanced if the study environment and technological aspects are strengthened by the college/university. The students should be allowed to use gadgets such as mobile phones/laptops on the college premises to support innovative FL as it stimulates self-study among the students to learn at their own pace and make use of their free time productively. Educators should facilitate and design learning experiences for tech-savvy students in this sustaining technology besides safe handling of gadgets without indulging in addiction to infuse FL. In the future, the study can be done on other nursing subjects and explored with nurse educators and administrators to assess their perception of FL.
